# IEEE 802.11af-enabled scalable cognitive radio sensor networks with adaptive priority management for early forest fire forewarning

**DOI:** 10.1038/s41598-025-21050-1

**Published:** 2025-10-23

**Authors:** V. Abilasha, A. Karthikeyan

**Affiliations:** School of Electronics Engineering, Vellore Institute of Technology, Vellore, India

**Keywords:** Cognitive radio sensor networks, Forest fire, IEEE 802.11af, Adaptive priority management, Emergency notification, Engineering, Environmental sciences, Natural hazards

## Abstract

Forest fires cause severe ecological and economic damage, and their early detection is critical for effective disaster management. Conventional wireless sensor networks often fail to deliver timely alerts during emergencies due to congestion and unreliable channel conditions in forest environments. This paper proposes a Cognitive Radio Sensor Network (CRSN) utilizing IEEE 802.11af technology for Forest Fire Early Warning and Emergency Notification System to address these challenges. The system integrates temperature, smoke, and gas sensors with Cognitive radio sensor nodes to detect forest fire events and prioritize emergency alert transmissions. By dynamically sensing and utilizing idle licensed channels, the system bypasses congestion, and an Adaptive Priority Management for prioritizing classes for emergency notification, ensuring low-latency and reliable delivery of fire alerts. Simulation results demonstrate that the proposed system achieves lower bit error rates and reduced latency under varying environmental conditions, enhancing the reliability and effectiveness of forest fire emergency notifications.

## Introduction

Forest monitoring systems are advancing rapidly due to achievements in technological innovation, wireless sensors, and wireless communication. It is now recommended to utilize sensor-based monitoring systems for effective forest management and fire prevention. The sensing interfaces of the monitoring equipment used in forest monitoring systems are user-friendly, adaptable, and efficient. These interfaces enable continuous monitoring of forest conditions within the natural environment. For long-term forest monitoring, the sensor deployment must remain flexible to accommodate evolving requirements, ensuring a smoother and more reliable monitoring process. Additionally, the system database includes the location information of monitored sites, allowing rapid emergency response and immediate notifications in the event of a forest fire or related emergency.

The network employs cognitive radio Wi-Fi based on IEEE 802.11af for dynamic spectrum access. This study utilizes IEEE 802.11af technology, commonly known as Wi-Fi over TV White Spaces (TVWS), to enable opportunistic, long-range wireless monitoring in forest environments. By leveraging Wi-Fi over TVWS, the system achieves cost-effective, spectrum-efficient forest fire detection without interfering with licensed primary services. A cognitive radio-based standard known as IEEE 802.11af technology enables the opportunistic and cost-free utilization of licensed broadcasting white spaces for forest monitoring applications. Cognitive radio sensor nodes (CRSNs), i.e., White Space Sensor Nodes equipped with TV White Spaces (TVWS), can leverage licensed television spectrum managed through a Cognitive Location Database (CLB) without causing interference to authorized operations in the area. The operating parameters of these (CRSNs), which function as opportunistic and unlicensed devices, can be adjusted based on their mobility within forest environments, whether deployed at fixed monitoring stations or mounted on mobile platforms such as drones or ranger vehicles. The CLB serves as the core of TVWS technology by storing authorized spectrum frequencies and CLB operational details linked to specific forest locations. With the support of intelligent detection devices utilizing TVWS, there is a significant opportunity to monitor environmental parameters and detect early signs of forest fires cost-effectively while ensuring reliable data tracking in diverse forest conditions.

The goal of this project is to develop a wireless sensor network with priority classes for forest fire monitoring using the TVWS methodology. This work aims to provide unlimited spectrum availability, enabling adaptive priority for forest fire monitoring with lower delay and utilization of energy. To prevent delays in the priority queue, a duration mechanism is employed based on the urgency of forest monitoring data. An abrupt rise in temperature and the detection of smoke within the forest may indicate the onset of a fire. Such critical data must be transmitted immediately to ensure timely alerts without occupying unnecessary priority queue time. Therefore, this type of data is transmitted with the highest priority using an adaptive priority-based solution, ensuring prompt and reliable delivery of forest fire alerts for rapid response and mitigation.

The Channel fading in Wireless transmissions in forested environments is influenced by multipath propagation, shadowing due to dense vegetation, and terrain irregularities. These effects can degrade the received signal quality, impacting spectrum sensing reliability and packet delivery. As highlighted in^1^, adaptive channel estimation and diversity schemes can help mitigate fading in such environments. The Atmospheric absorption in Environmental conditions, such as humidity, rainfall, and gas concentration in forests, also causes frequency-selective attenuation. As shown in^2^, absorption phenomena become significant at higher frequencies, potentially impacting long-range CRSN communication.

The proposed CRSN leverages TV White Spaces under IEEE 802.11af, where absorption is relatively lower compared to millimeter-wave bands, fading and absorption remain non-negligible factors. To address this, the method integrates channel availability queries and link confirmation mechanisms, which dynamically adapt the spectrum allocation to maintain reliable communication even under adverse conditions.

The importance of this work lies in the use of an Adaptive Priority Management for prioritizing classes for emergency notification, such as forest fire alerts in smart forest monitoring based on cognitive radio. The cognitive radio sensor network makes opportunistic use of the spectrum without incurring costs, which is crucial for enabling scalable and low-cost forest monitoring applications. The primary contributions of this work include:

1. Forest monitoring is conducted using sensor networks with the Adaptive Priority TVWS methodology
2. Licensed television white spectrum is utilized without incurring any spectrum fees
3. Depending on the urgency, priority classifications are employed as level 1 (fire alerts), level 2 (potential risk indicators), and level 3 (routine monitoring data)
4. An Adaptive Priority Management is applied based on the urgency of forest monitoring data to prevent lower-priority data starvation
5. Graphs show that the suggested network is accurate and feasible, and
6. Adaptive Priority data tracking improves the efficacy of cognitive radio sensor networks for emergency alerting and forest fire detection on the TV white space spectrum.

## Related works

A reinforcement learning-based routing protocol^3^ for SDN-enabled wireless sensor networks targeting forest fire detection, enhancing routing efficiency and adaptability under dynamic forest conditions. It demonstrates improved detection accuracy, reduced delay, and optimized energy usage, making it suitable for scalable and reliable forest fire monitoring. Dynamic fuzzy-based main user awareness is a novel approach to cognitive radio sensor networks presented in the study^4^. It employs a dynamic method to determine the number of clusters, a fuzzy-based methodology to select the best cluster head, and dependable multi-hop data transfer to secure the primary user.

To mitigate the impact of faulty spectrum sensing on network performance^5^, proposed a multi-hop clustered routing algorithm that leverages imperfect spectrum sensing. To choose cluster heads as well as relays that have significant spectrum sensing capabilities, selection criteria are established based on the detection-level function of the channels available. The functionality provided by a common cognitive radio sensor network featuring simultaneous wireless power and information sharing on extended^6^ Nakagami fading channels is given. For this, a time-shifting protocol was used to account for the influence on the energy collecting, information transfer, data processing, and broadcasting phases.

With an emphasis on the crucial component of spectrum sensing^7^, introduced an energy optimization technique tailored for CRWSN. For field hospitals, the Cognitive Radio Sensor Network^8^, enables priority-based data transmission during emergencies without spectrum cost by leveraging cognitive radio capabilities. It demonstrates improved reliability, reduced latency, and effective spectrum utilization, ensuring the timely delivery of critical patient and operational data.

A primary sensor with a secondary sensor is part of the two-stage detection strategy employed in the suggested technique. In order to maximize the reporting time and effectiveness of cognitive wireless sensor networks^9^, suggested an efficient collaborative spectrum sensing method that consists of data fusion, multibit quantization, selective reporting, and dynamic parameters. Sensor nodes first selectively report some essential observations before encoding their findings using multibit binary codes while transmitting them to the fusion center. The technique for several arbitrary primary user and secondary user applications was described in^10^. Their suggested algorithm raised the convergence speed in comparison to the current optimization techniques by integrating multiuser clustered communication.

By framing position assessment as a multicriteria group decision-making problem^11^, introduced a novel algorithm, then gave both a direct and an indirect presentation of the ideas of fuzzy sets and set pair analysis. Cognitive sensor network for smart agriculture^12^, using priority-based data handling to detect and alert critical events like pest attacks or drought stress efficiently. It leverages cognitive radio for opportunistic spectrum access, ensuring low-latency, cost-effective emergency alerts without interfering with licensed users.

A QoS-aware green cooperative compressed sensing strategy was discussed in^13^ in order to optimize DA-WRAN’s energy efficiency while adhering to its fundamental QoS constraints of packet failure probability, sensing delay, and overall throughput. They also brought compressed sensing theory into DA-WRAN. For agricultural monitoring^14^, suggested a sensor network based on cognitive wireless regional area network (WRAN) technology. With the use of Internet of Things gear and software^15^, they were able to produce a successful corn harvest on large-scale fields.

A new clustering technique^16^ for WSNs was proposed to lower energy usage and increase the WSNs’ lifespan. Tao et al.^17^ provided a critical summary of the most recent studies relevant to IoT connectivity technology and smart agriculture. Initially introduced in^18^, the IEEE 802.11af standard offers universal norms for spectrum utilization among unlicensed white-field devices, including licensed services running in the television white-field range. Complementary metal–oxide–semiconductor (CMOS) transmitters for IEEE 802.11af wireless local area networks have been used to create a unique composite distortion^19^ correcting the digital pre-distorter. A wireless transmitter with a multi-class linearized power amplifier and IEEE 802.11af^20^ compliance is provided for wireless local area networks. The comparative analysis presented in Table 1 highlights the key differences between the proposed adaptive priority management mechanism and other methods.

**Table 1:** Comparison analysis of adaptive priority management and other techniques.

Feature/Advantage	Adaptive Priority Management with FDMA	PSP^22^	APD-GRED^23^	ASMF^21^	Cognitive Sensor Network^12^
Dynamic Context-Aware Scheduling	Adjusts based on network load, urgency, and wait time	Preemptive scheduling without dynamic context adaptation	Queue-based differentiation without environment-awareness	Focuses on spectrum adaptation, not scheduling logic	Priority not dynamically adjusted
Low-Latency Communication	Uses FDMA to prevent collisions and reduce delays	Not optimized for latency-critical transmissions	Focused on fairness, not latency	Emphasis on fault tolerance, not real-time speed	Not explicitly designed for emergency low latency
Energy Efficiency	Idle-mode operation, optimized transmissions	No explicit energy-saving design	Not addressed	Includes energy-efficient communication	Not addressed
Fairness & Starvation Prevention	Promotes older packets to avoid starvation while preserving urgency	Focuses only on high-priority users	Provides fairness across queues but lacks adaptive promotion	Not explicitly addressed	Not addressed
Regulatory Compliance (TVWS)	Ensures authorized spectrum use via LAS & CLB	Not discussed	Not discussed	Not applicable	Not discussed
Scalability	Designed for large-scale forest monitoring networks	Evaluated in small-scale CRNs	Tested in controlled settings	Limited scalability insights	Not addressed
Robustness in Harsh Environments	Handles node failures, energy constraints, and channel fluctuations	Not considered	Not considered	Fault-tolerant design, but not tailored for forest conditions	Not addressed

### Design of TV white spaces (TVWS)

The present work leverages television white spaces for Adaptive priority management for forest surveillance using the IEEE 802.11af standard. This enables the opportunistic utilization of an integrated suite of services at no cost, while ensuring that Cognitive radio Wireless Sensor nodes do not interfere with protected primary services due to TVWS’s regulatory domains and structured spectrum allocation.

The choice of IEEE 802.11af in this work is deliberate and grounded in the specific requirements of forest surveillance and fire monitoring applications, as elaborated below:

- Opportunistic Utilization of TV White Spaces with Structured Regulatory Support
- Support for Dense, Energy-Constrained Sensor Deployments
- Moderate Range with Higher Data Rates Suitable for Environmental Monitoring
- Dynamic Channel Management and Reliability in Harsh Conditions
- Low-Latency Priority-Based Communication through FDMA
- Scalable, Multi-Hop Ad Hoc Routing for Extended Coverage

**Fig. 1:**
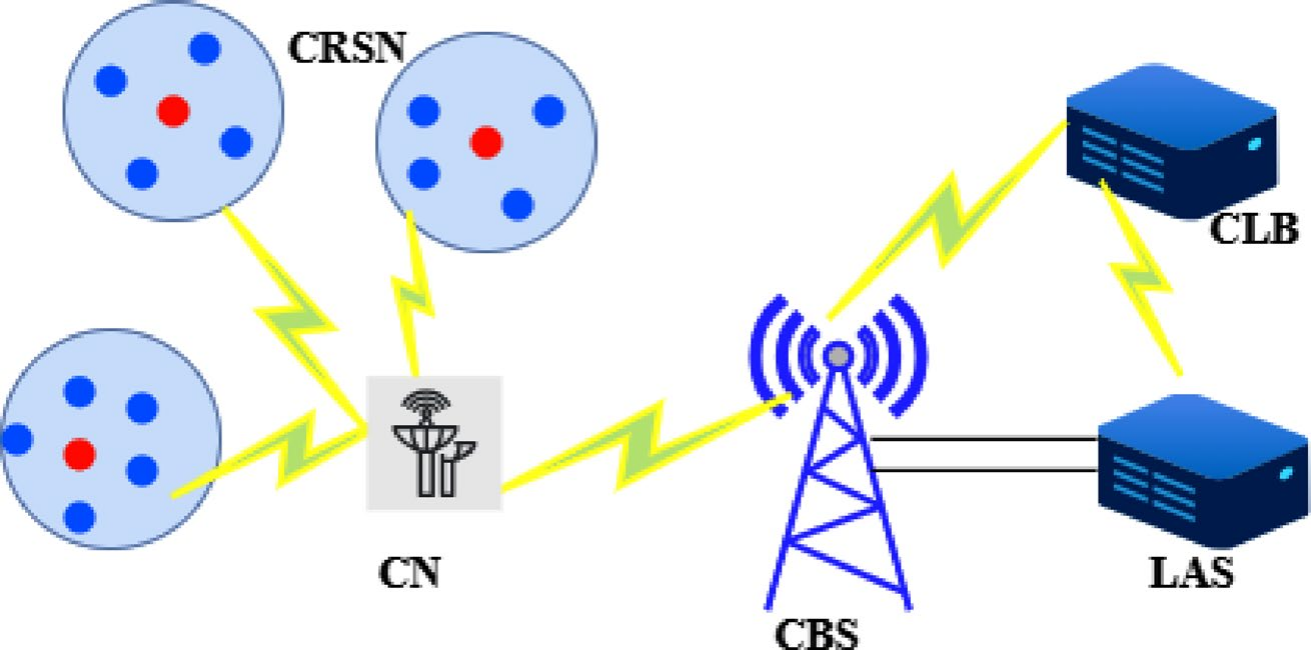
System architecture of IEEE 802.11af.

Figure 1 illustrates the IEEE 802.11af-based priority forest monitoring setup. The wireless network environment consists of CRSNs, Cognitive Base Station (CBS), Cognitive Node (CN), a Location Authentication Server (LAS), and a Cognitive Location Database (CLB). The CLB stores the authorized spectrum frequencies and operational parameters, ensuring that CRSNs adhere to regional compliance requirements.

The LAS and CLB can communicate directly over a wireless link, and the CBS can also communicate directly with the CLB via Wi-Fi. Wireless exchanges occur between CRSNs, CHs, and the CBS. The CH, managed by the CBS and connected to the CLB, receives authorized spectrum frequencies and parameters as Coordination Information (CI) from either the CN or the LAS.

Additionally, sensor devices can be deployed underground within the proposed network architecture, where temperature sensors measure temperature, humidity sensors capture humidity, and pressure sensors detect pressure. Other environmental parameters can also be monitored using appropriate sensors. The CRSNs collect all sensed data and transmit it to any internet-connected device, enabling authorities to monitor forest conditions in real time.

### Channel management

Using the channel availability query, the Cognitive Base Station (CBS) retrieves the available spectrum frequencies in TVWS Channel Map (TVWSCM) format at its current forest location. Information about channel availability is provided promptly either through the Location Authentication Server (LAS) or by accessing the Cognitive Location Database (CLB) to support the CH.

A channel availability query within the network structure can be initiated under various conditions to ensure reliable data transmission in forest monitoring:

1. When the CH remains in the activation state after a timeout while awaiting channel allocation;
2. When a Primary User (PU) is detected or there is interference in the present channel, the CBS indicates an alteration in channel availability and
3. When the CH moves beyond its permitted operational range within the monitored forest

The information regarding the white space is acquired by the CBS through channel management procedures to support seamless communication. The CBS requests channel schedule management from the LAS to determine which television channels are available for opportunistic use, ensuring that the CH in the forest monitoring system does not need to individually manage channel scheduling for white space channels in the wireless sensor network for cognitive radio.

### Link confirmation message

The CBS transmits the link confirmation message in the forest monitoring system for two primary purposes:

1. To detect new sensor nodes entering its service area within the monitored region and
2. To ensure that the nodes are operating under a valid TVWSCM.

The Algorithm 1 for Link confirmation message operates as follows:

Initially, the CBS acquires the Channel Reference Tag (CRT) associated with the Link confirmation message. It then checks whether this CRT matches the current CI. If the CRT matches, the operating CI is considered valid, and the CBS resets the verification timer after a timeout period. The system then verifies the arrival of a new Link confirmation message. If no new signal is received, the current CI remains valid, allowing continued use of the TVWSCM for forest monitoring data transmission. If a new signal is received, the current CI is updated using the CRT from the new signal.

**Figure:**
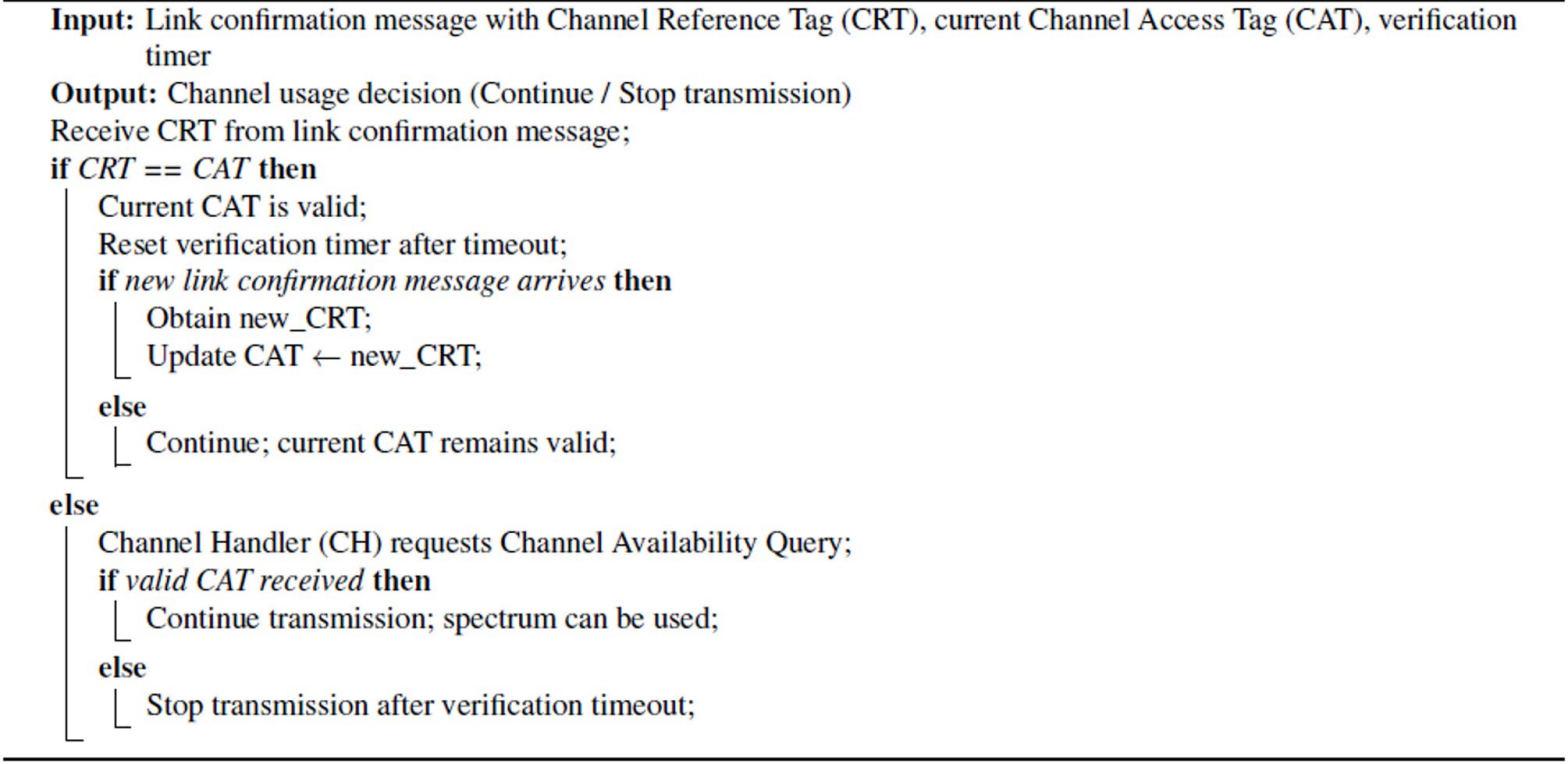
Algorithm 1 Link confirmation message-verification signal handling using channel access tag

If the CRT does not match the current CI, the CH initiates a channel availability query for the current CI to verify the usable spectrum at its location in the forest. If a valid CI is not received within the validation period, transmission is halted to prevent interference and ensure compliance with spectrum regulations. However, if a valid CI is acquired through the fresh channel availability query, the system confirms the reliability of the operational CI, allowing continued, interference-free use of the TVWS for adaptive priority-based forest fire monitoring and emergency alert transmission.

### Location database reliance

Through the Location Database Reliance (LDR) enabling process, the CBS can establish a forest monitoring network under the authority of the CLB in compliance with regulatory requirements. To provide the LDR activation service, the CBS transmits a beacon signal across the available television white space channels within the forest monitoring area. Upon receiving the LDR activation signal, the CH initiates the activation process using the LDR initiation response frame, allowing it to operate within the authorized white space spectrum for continuous and compliant forest fire monitoring and emergency alert transmission.

#### TVWS cognitive radio sensor network for forest fire monitoring

The presented TVWS cognitive radio sensor network’s simulation settings can be used for adaptive priority management in forest fire monitoring without needing spectrum expenditures, for CRSNs, Cognitive node, and CBS, which were calculated. Table 2 illustrates the suggested sensor network design’s simulation settings. Each simulation parameter is defined independently because some of them are bidirectional.

**Table 2:** Simulation parameters.

Parameter	Range
Area	200 m × 200 m
Coverage distance	250 m
Path loss coefficient	4
SNR	8 decibels
Transmission power	40 mw
Operating frequency	2437 MHz
Bandwidth	20 MHz
Data rate upload	200 Kbps
Data rate download	1000 Kbps
Modulation scheme	QPSK
Data packet size	128 bytes

**Fig. 2:**
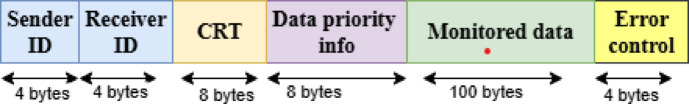
Structure of the packet.

Figure 2 shows the packet structure used in the system. A packet’s total size is 128 bytes and includes the sender ID, receiver ID, CRT, data priority, monitored sensor information, and error control codes. The CRT indicates the relevant TVWSCM, and the priority class of the corresponding sensor data is labeled as either level 1, level 2, and level 3, subject to how critical the data from forest monitoring is. Priority levels are defined with level 1 as the highest and level 3 as the lowest priority.

In this system, forest sensors measure temperature, smoke, and gas concentrations to detect early signs of forest fires.

#### Design of adaptive priority management in cognitive radio sensor network 

Cognitive Radio Sensor Network utilizing Adaptive Priority Management for forest fire monitoring includes sensor devices, TCWSCM, Cognitive Nodes (CN), and Cognitive Base Station (CBS) that interact using Frequency Division Multiple Access (FDMA). FDMA, being a contention-free technology, eliminates delays caused by packet collisions, which is crucial during emergency forest fire alerts. The FDMA approach prevents packet collisions, reduces energy consumption, and maximizes transmission efficiency across the forest monitoring network.

In this system, TVWSCM collects data from forest sensors measuring temperature, smoke, and gas concentrations to detect early signs of forest fires. The vitality of the noticed data impacts its inclusion of priority details in the packet. The network employs a barrier-free Poisson-General-Single Server (P/G/1) priority queuing system, where packets with equal priority levels follow a first-in, first-out (FIFO) procedure to ensure fairness while prioritizing urgent alerts.

Once the TVWSCM collects the data, it transfers the packet to the CN, which then sends it to the CBS for further processing. Authorities can access all data received by the CBS using online devices for real-time monitoring and rapid response in the event of a forest fire.

The priorities of the monitored data are based on urgency, with level 1 indicating the highest priority for critical fire alerts, level 2 indicating potential risk conditions, and level 3 representing routine environmental monitoring data. CBS, CNs, and TVWSCM are made possible by the FDMA frame structure, which splits the band’s spectrum into distinct channels for a given channel capacity of 100 kHz, to transmit and receive data on any allocated channel without collisions, ensuring efficient and reliable communication essential for low-latency forest fire emergency notification.

Adaptive priority management introduces several key differences:

- *Adaptive Reassignment*: Packets that remain in the queue for more than three frame periods are automatically promoted to higher priority levels, ensuring fairness and preventing starvation.
- *Age-Sensitive Scheduling*: Older packets within the same priority class are transmitted first, thereby reducing excessive waiting time.
- *Fairness with Emergency Assurance*: New packets of equal priority are pushed to the back of the queue (FCFS), balancing fairness with responsiveness.
- *Contention-Free FDMA Integration*: Unlike existing queuing methods, our system integrates adaptive priority with FDMA-based contention-free scheduling, eliminating packet collisions while ensuring low-latency emergency delivery.
- *Regulation-Compliant Spectrum Management*: By operating under IEEE 802.11af with CLB and LAS authorization, our adaptive mechanism ensures TVWS-compliant spectrum access without additional spectrum expenditure—an aspect largely absent in conventional models.

*Service Time*: The service time (T_s_) represents the duration required to transmit one data packet over an allocated frequency channel. The FDMA approach expresses each packet’s service time in the forest fire monitoring CRSN as:

$${T}_{s}=\frac{N{L}_{p}}{{D}_{r}}$$

where, T_s_ is the service time, N gives the number of active CRSNs in the forest monitoring network, L_p_ specifies packet length and D_r_ denotes the data rate.

In FDMA, each active node is assigned a distinct frequency channel to avoid collisions. Thus, N represents the total number of nodes sharing the available spectrum. The total load is proportional to the number of nodes and the size of their packets, while inversely proportional to the data rate, representing the time required to serve one packet.

*Total Load Estimation*: The offered payload and T_s_ represent the timeslot duration for all successful transactions within a frame. The total payload in the FDMA system is estimated as:

$$\rho = \lambda {\text{T}}_{{\text{s}}}$$

where, $\rho$ provides the total load, λ denotes the packet generation rate by CRSNs following a Poisson process, and T_s_ is the service period.

This formula reflects how often the method is being used relative to its capacity and helps in analyzing how close the network operates to saturation or congestion.

*System Throughput Expression*: The performance equation for FDMA technology in the forest fire CRSN is expressed as:

$${\text{Y}} = \rho {\text{N}}$$

where, Y provides the system performance, $\rho$ is the total load, and N denotes the total number of active CRSN.

FDMA, being contention-free, ensures uninterrupted transmissions across channels, crucial for timely forest fire emergency alerts. This gives the aggregate throughput of the network, representing how much data can be transmitted collectively across all active nodes.

*Delay Derivation Using the P/G/1 Queuing Model*: The estimated delay (${\tau }_{d}$) for a packet using the P/G/1 queuing system in the forest fire monitoring scenario is given by:

- The P/G/1 model accounts for a general service time distribution and Poisson packet arrival.
- The numerator reflects the combined effect of packet service time and arrival rate fluctuations.
- The denominator ensures that the system remains stable as long as 2 − 2λTs > 0, i.e., the system is not overloaded.
- This form clearly shows how the service time and total load impact the delay.
- As ρ approaches 1, the denominator approaches zero, reflecting that delay increases significantly under high load conditions.
  $${\tau }_{d}=\frac{2{T}_{s}-2\lambda {T}_{s}^{2}+\lambda {T}_{s}^{2}}{2-2\lambda {T}_{s}}$$

By replacing ρ = λ T_s_, the anticipated packet delay becomes:

$${\tau }_{d}=\frac{{T}_{s}\left(2-\rho \right)}{2\left(1-\rho \right)}$$

This form clearly shows how the service time and total load impact the delay. As ρ approaches 1, the denominator approaches zero, reflecting that delay increases significantly under high load conditions. Alternatively, by using Y/ N in place of ρ:

$${\tau }_{d}=\frac{{T}_{s}}{2}\left(1+\frac{N}{N-Y}\right)$$

This expression connects throughput (Y) with delay (${\tau }_{d}$​), offering insights into how close the system is to congestion. It demonstrates that as Y approaches N, the delay grows rapidly, reinforcing the need for priority management to ensure timely data transmission during emergencies. These formulations allow the analysis of throughput and delay characteristics under a fixed packet size in FDMA-based forest fire CRSN monitoring.

Algorithm 2 can be followed as:

- Each CRSN is assigned a clear channel before initiating packet transfers.
- Guard bands are added between channels to prevent inter-channel interference.
- The priority queue is checked in the following order:
  - Level 1 (High Priority) packets are transmitted first, ensuring immediate forest fire alerts.
  - If no level 1 packets are present, level 2 (Medium Priority) packets are checked and transmitted if available.
  - If neither level 1 nor level 2 packets are present, level 3(Low Priority) packets are transmitted for routine environmental monitoring.

Algorithm 2 shows the priority-based FDMA approach in forest fire monitoring.

**Figure:**
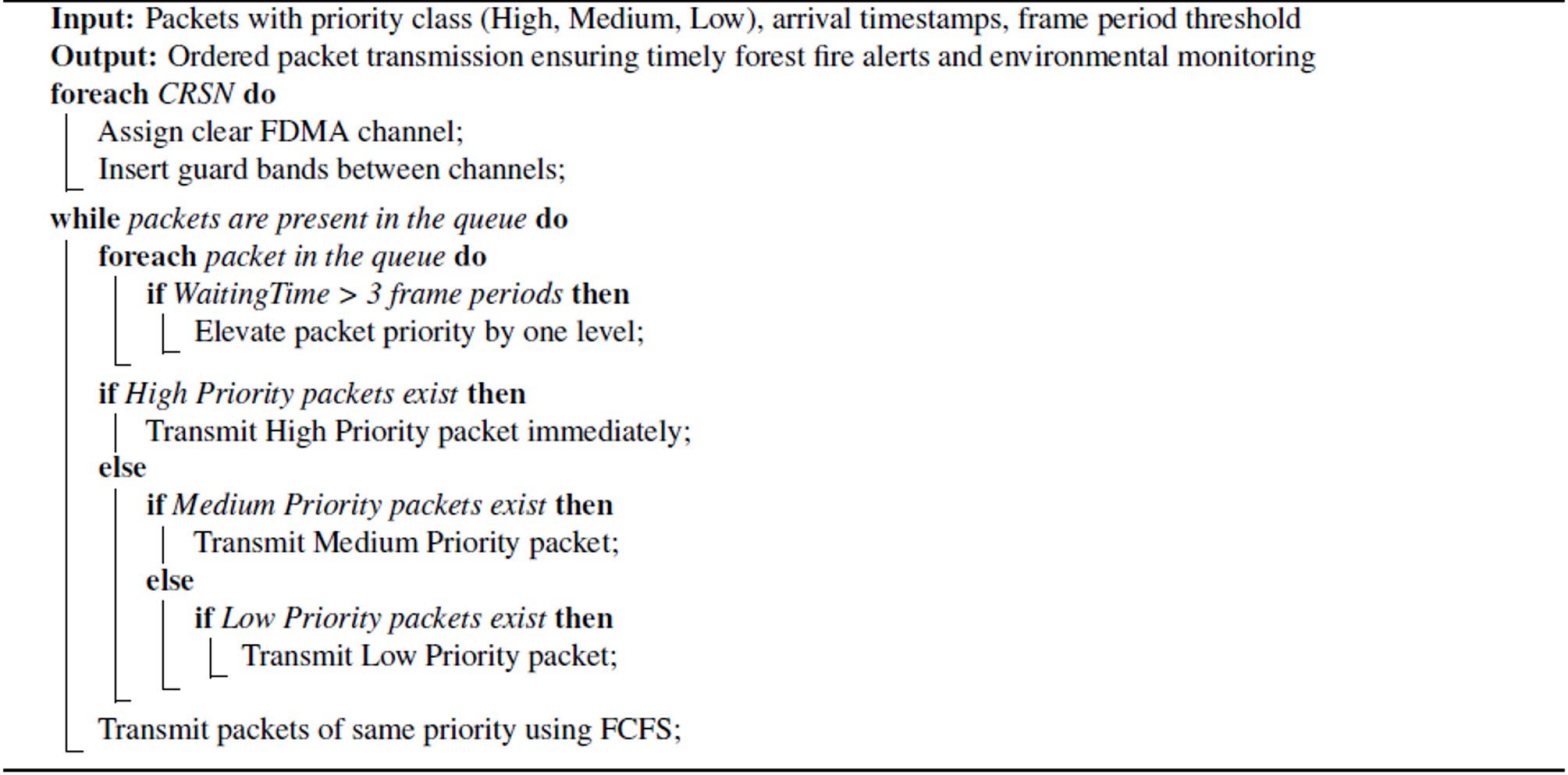
Algorithm 2 Priority-based FDMA Packet transmission with waiting-time-based priority elevation for forest fire monitoring

This cycle repeats until all packets are transmitted.

In high arrival rate conditions, low-priority packets may experience starvation due to continuous high-priority packet arrivals. To address this, an Adaptive priority management is implemented:

- Packets that remain in the queue for more than three frame periods are given higher priority.
- Older packets with the same priority are transmitted before newer packets, reducing excessive wait times.
- New packets with equivalent priority are pushed to the back of the queue using First-Come-First-Served (FCFS) discipline.

**Fig. 3:**
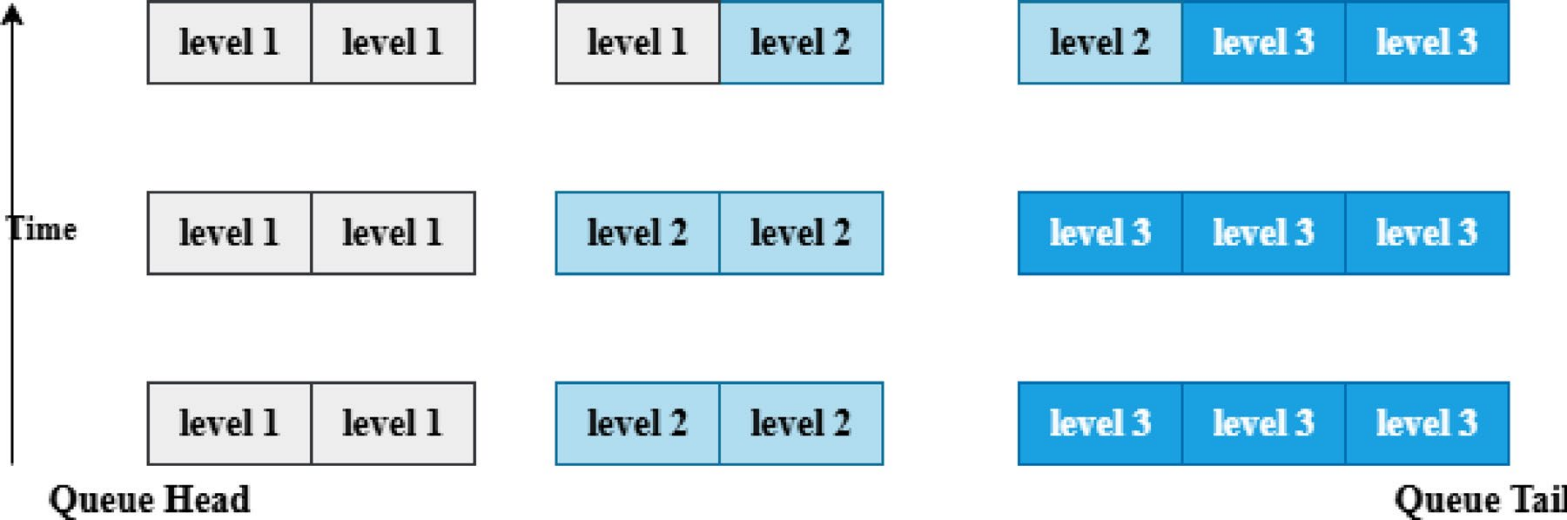
Adaptive priority management.

For example, a sudden spike in temperature or smoke detection indicating a possible forest fire requires immediate level 1 priority transmission to prevent delays in the alert system, even when adaptive priority is applied. Figure 3 demonstrates how duration ensures that packets do not experience starvation, preserving low-latency emergency communication in the forest fire CRSN.

### Simulation model setup

The cognitive radio sensor network (CRSN) for forest fire monitoring is simulated using MATLAB software. The assessment of outcomes regarding the proposed network over various emergencies and forest conditions is made possible by discrete event simulations.

There are three crucial steps in configuring the wireless sensor network simulation:

*Network Stage*: The topology of the forest monitoring CRSN is established, defining the spatial distribution of Cognitive nodes (CNs), Cognitive Radio Sensor Nodes (CRSNs), and Cognitive Base Station (CBS) across the forest area for effective coverage and connectivity.

*Node Stage*: The behavior of each node is modeled, including sensing, spectrum sensing, channel access, and priority-based packet generation and queuing (level 1, level 2, level 3) based on detected temperature, smoke, and gas concentration data in the forest environment.

*Transaction Stage*: This stage manages the transmission, queuing, and scheduling of packets using FDMA with priority mechanisms under dynamic forest conditions. Performance parameters such as latency, throughput, energy consumption, and bit error rate (BER) are monitored to assess the system’s efficiency and reliability during forest fire alerts.

The state machine behavior of sensor nodes and CRSNs is implemented in MATLAB using structured scripts and function-based modular simulation blocks. States and transitions, such as sensing, idle, transmitting, and spectrum handoff (when a primary user appears), are systematically handled to reflect realistic operational scenarios during forest fire detection and emergency notification.

**Fig. 4:**
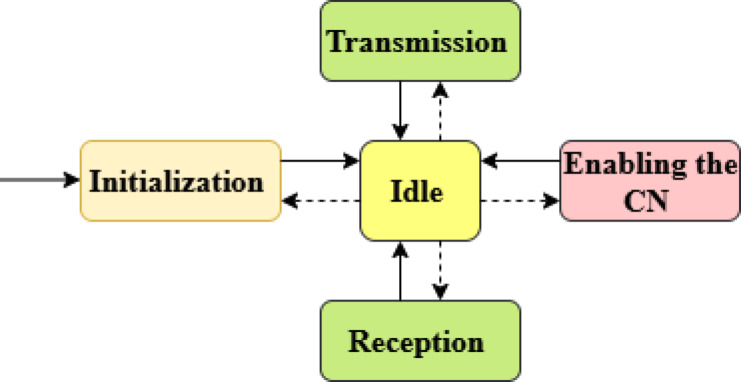
Operation model of the CN.

After defining all simulation variables, initial values are assigned in the initialization state. Figure 4 shows the operation model of the CN. During the idle state, CN waits for the next event trigger. Upon receiving the location Database Reliance (LDR) activation signal, the LDR activation process begins, enabling the CN to operate on authorized white space frequencies.

During the receiving state, the CN uses the FDMA mechanism to receive high-priority fire alert packets and monitoring data from CRSNs. In the transmission state, FDMA-based channel planning is used to forward the received packets to the CBS, prioritizing packets based on their urgency (level 1, level 2, level 3) to ensure low-latency emergency communication during forest fire events. During the data collection phase, the CRSN gathers data from forest sensors measuring temperature, smoke, and gas concentrations for early fire detection.

**Fig. 5:**
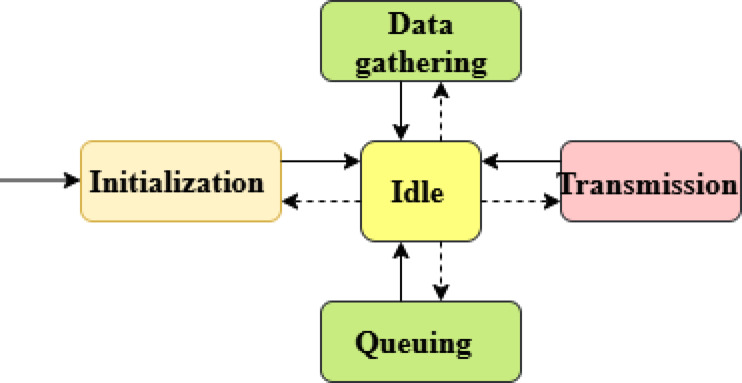
Operation model of the CRSN.

Figure 5 shows the operation model of the CRSN. When the queuing state is active, the collected sensor data is encapsulated into packets along with priority information, which are then stored in the transmission queue. During the transmission phase, packets are sent to the CN using FDMA-based channel planning, ensuring that high-priority alerts (level 1) are transmitted first, followed by level 2 and level 3 packets as per available channel capacity. In the idle state, the CBS awaits event triggers. Using LAS and the CLB, the CBS manages TVWSCM dynamically for effective spectrum use in the forest monitoring environment.

When the Link Confirmation Message (LCM) is active, it is used both to identify CNs within the coverage area and to verify that CNs are operating under valid TVWSCM configurations. In the queuing state, the CBS organizes received packets based on priority classifications before processing. In the reception state, CNs deliver packets to the CBS following FDMA-based channel allocation, ensuring uninterrupted priority-based data reception during forest fire monitoring.

### Primary user activity

The simulation integrates primary user (PU) activity patterns by dynamically altering channel availability within the TVWS Channel Map (TVWSCM). The PU is modeled as intermittently occupying channels based on a stochastic process, where the Channels are marked “busy” when PU signals are detected, causing the CBS and CRSNs to query the CLB for alternate available channels. A timeout mechanism is applied during channel allocation to ensure nodes respond to changes in PU presence. Interference scenarios are explicitly simulated by triggering PU activity during active data transmission periods, forcing channel handoffs, and validating the robustness of the adaptive priority mechanism.

#### Mobility scenarios

While forest monitoring nodes are primarily stationary, the simulation includes mobility patterns for Cognitive Nodes, which are implemented as follows:

- Random waypoint and linear movement patterns are modeled within the defined coverage area (200 m × 200 m).
- When nodes move beyond their operational range, channel availability queries are initiated to reassign valid spectrum resources.
- Mobility-triggered reconfiguration is validated by the link confirmation message protocol and the Location Database Reliance (LDR) activation procedure.

This ensures that both stationary and mobile nodes operate reliably in dynamic forest environments.

To validate our simulation results, we compare the proposed CRSN setup with:

#### A baseline conventional wireless sensor network (WSN)

- Uses fixed spectrum allocation without dynamic channel management or opportunistic access.
- Serves as a control case to evaluate the benefits of priority-based FDMA transmission and energy efficiency.

#### A non-priority CRSN implementation

- Employs cognitive radio features such as TVWS access but lacks adaptive priority handling and channel availability queries.
- This allows us to quantify improvements in latency, throughput, and energy consumption due to priority-based scheduling and link confirmation protocols.

Simulation metrics such as latency (τ_d_), throughput (Y), and energy consumption are benchmarked across these scenarios, demonstrating the superior performance of the proposed method, particularly in maintaining uninterrupted data flow during emergency forest fire alerts.

#### During simulation runtime in MATLAB

- Data packets are generated using a Poisson process to model realistic forest sensor data generation.
- Sensor nodes that are not in direct communication range with the CBS use multi-hop ad hoc routing through neighboring nodes to relay sensed data. The CBS stores all received sensed data for analysis and emergency notifications.
- Unlicensed sensor nodes enter sleep mode when idle to minimize energy consumption during long-term monitoring.
- The First-Come-First-Serve (FCFS) algorithm is applied to handle newly arriving packets of the same priority. CRC are used for Error control codes.

## Results and discussion

**Fig. 6:**
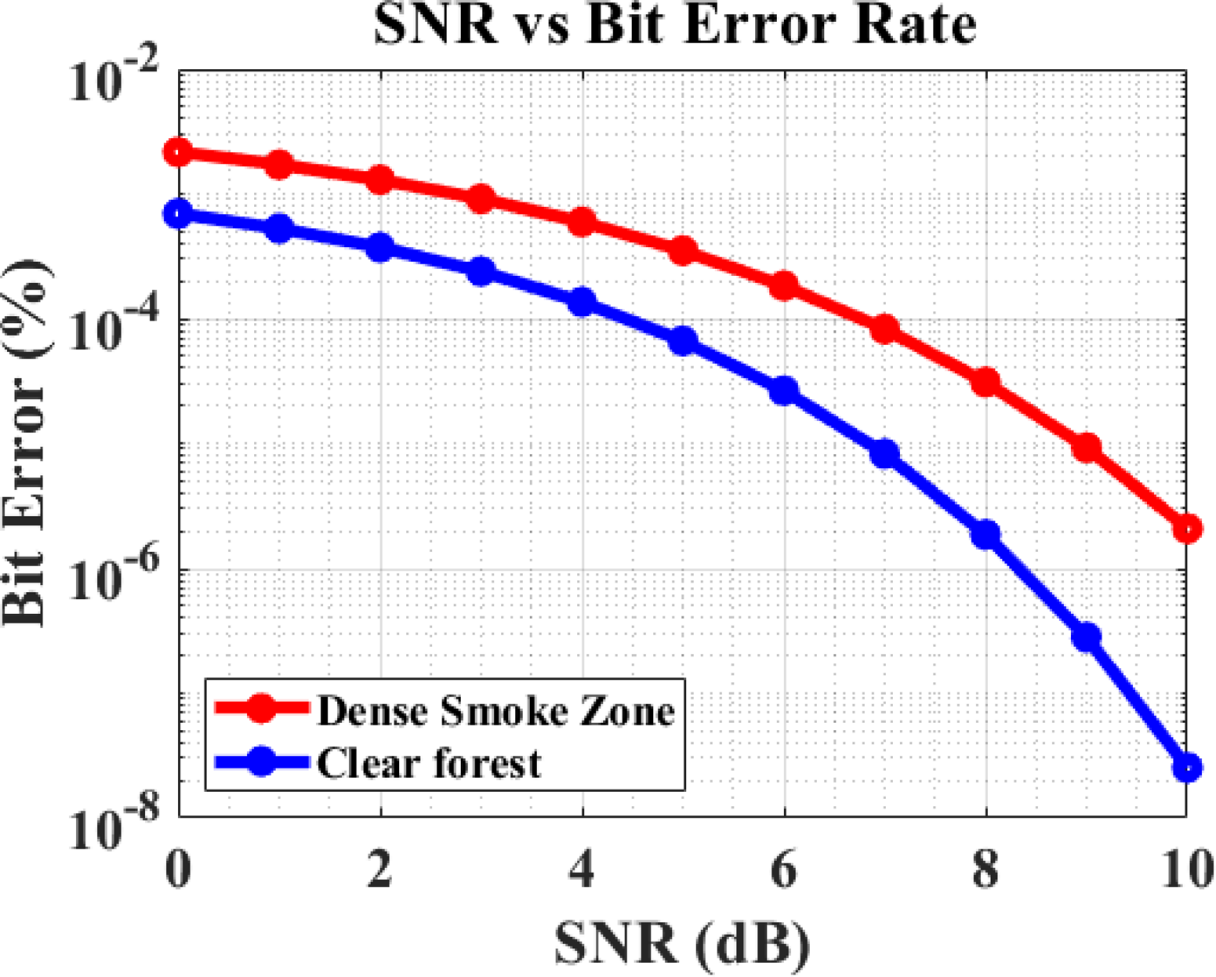
SNR VS BER.

The proposed forest fire cognitive radio sensor network (CRSN) was evaluated using MATLAB simulations based on the following key performance metrics. Figure 6 displays the bit error rate (BER) results for communication in both dense and sparse forest environments based on the signal-to-noise ratio (SNR). The BER was computed by dividing the total number of bits sent over a specified simulation period by the number of bit errors detected during transmission. Minimizing the BER is crucial for optimal network performance, ensuring reliable forest fire detection and emergency alerts even in challenging environmental conditions.

### Under varying SNR conditions

*Clear Forest environments exhibit lower BER* compared to dense forest environments due to reduced multipath fading and lower signal attenuation.

*Dense Forest environments exhibit higher BER* due to the dampening and scattering effects caused by foliage density, moisture content, and terrain irregularities, which degrade signal quality during transmission.

**Fig. 7:**
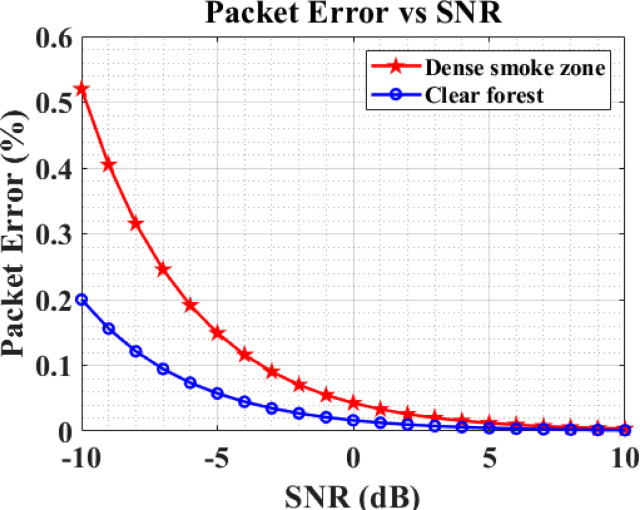
SNR VS PER.

Figure 7 displays the packet error rate (PER) results for sensor transmissions in both dense and clear forest environments based on the signal-to-noise ratio (SNR). The PER was computed by dividing the number of incorrectly received packets by the total number of packets transmitted over a specified simulation period.

Minimizing the PER is essential for improving network performance, ensuring reliable transmission of critical forest fire alerts and sensor data under varying environmental conditions.

For an equivalent SNR of up to 6 dB, sensors deployed in dense forest environments exhibit a higher PER compared to those in sparse forest environments. This is due to the attenuation, scattering, and multipath fading effects present in dense forests, which degrade signal quality and increase the likelihood of packet errors during transmission.

Beyond 6 dB SNR, the PER for sensors in both dense and sparse forest environments converges, indicating that higher SNR levels can overcome the environmental attenuation effects, resulting in reliable packet delivery irrespective of forest density.

These PER findings validate that environmental conditions in forest monitoring directly impact network reliability and that the proposed forest fire CRSN framework accurately captures these effects, aiding in the design of robust emergency notification systems for real-world deployment.

**Fig. 8:**
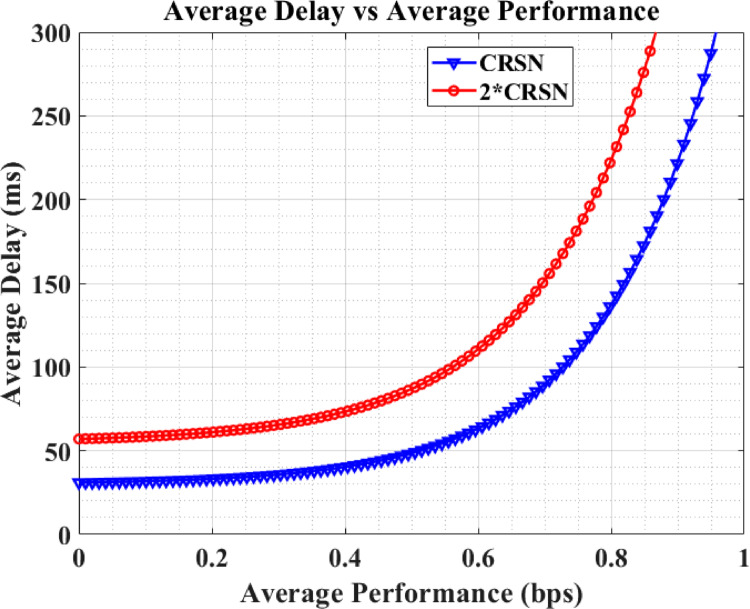
Average performance vs delay.

Figure 8 displays the performance–latency characteristics for CRSNs and the performance curve when the number of CRSNs in the forest monitoring network is doubled.

Average latency represents the time taken for critical fire detection data to travel across the network from sensor devices to the CBS.

- Average performance reflects the successful packet transmission rate between nodes in the CRSN.

When the number of CRSNs is doubled, the average latency increases slightly while the average performance experiences a marginal decline. This indicates that the chosen number of CRSNs provides a suitable balance between coverage and network load without significantly degrading performance, ensuring timely forest fire emergency alerts.

**Fig. 9:**
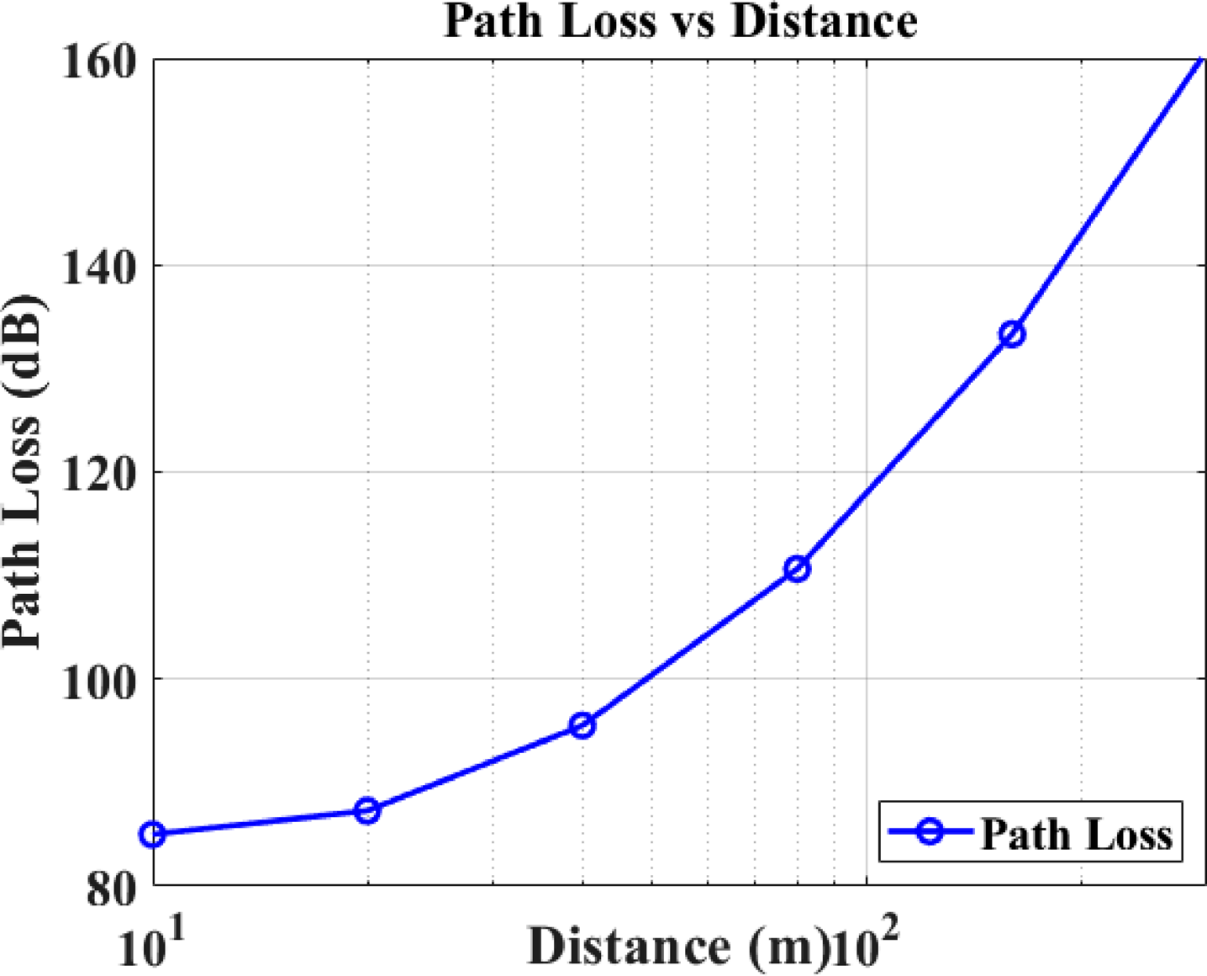
Distance versus path loss.

Figure 9 displays the path loss results by distance for the forest environment, indicating maximum coverage capabilities. Path loss, defined as the reduction in power density of an electromagnetic wave as it propagates through the forest environment, is a critical factor in analyzing and designing the communication link budget for the CRSN.

As the distance increases from 0 to 150 m, the path loss increases from 90 to 110 dB, demonstrating the attenuation effects due to foliage density, terrain, and moisture content. These results confirm the effective operational range of the proposed forest fire CRSN for reliable data transmission during fire monitoring.

**Fig. 10:**
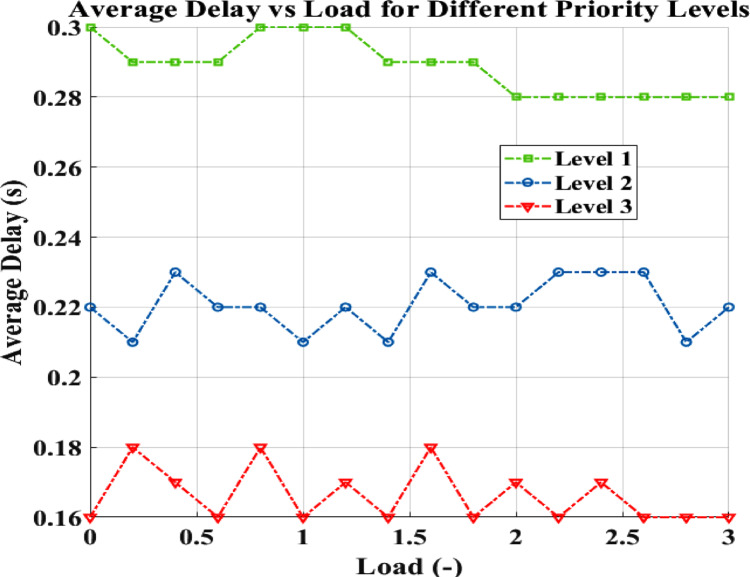
Load versus average delay.

Figure 10 displays the average delay results under varying network load conditions.

As network load increases, average delay increases due to higher queuing and transmission times within the priority-based FDMA structure. Despite this, the system maintains acceptable latency levels for high-priority level 1 packets, ensuring prompt fire emergency notifications even under heavy network usage, which is critical for early fire detection and response in forest environments.

The network load in the forest fire CRSN simulation refers to the number of data packets detected and generated by a sensor node per second during forest monitoring and fire detection activities.

**Fig. 11:**
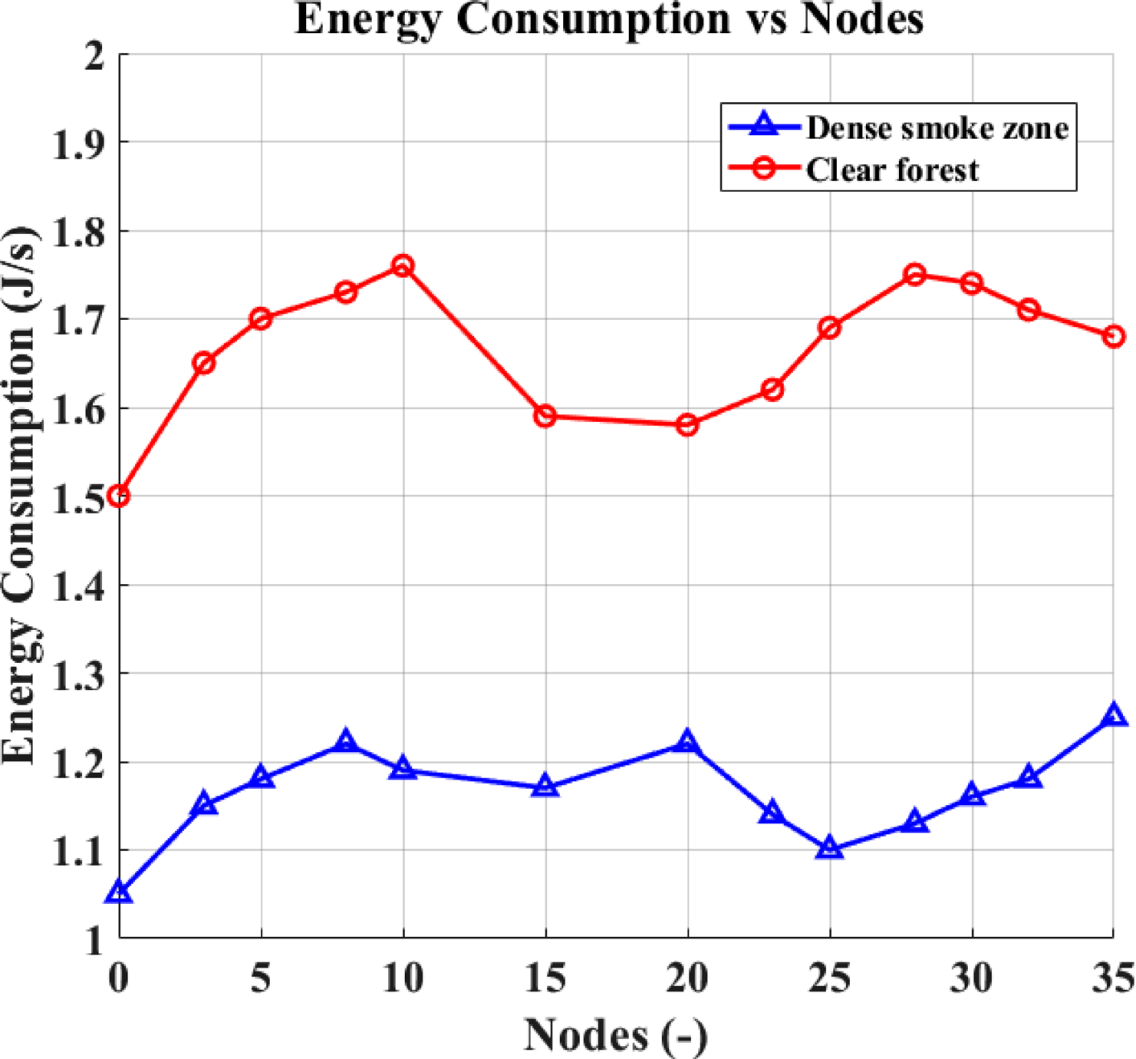
Nodes versus energy consumption.

Figure 11 displays the energy consumption results under dense and clear forest environments based on the number of deployed sensors. Energy consumption is defined as the total amount of energy utilized by the network, encompassing sensing, processing, idle, and transmission operations of the CRSNs, CNs, and CBS during forest fire monitoring.

### Under equivalent deployment conditions

Dense forest environments show higher energy consumption compared to clear forest environments due to the increased transmission power required to overcome foliage attenuation and maintain connectivity, as well as the need for multi-hop forwarding in obstructed terrains.

As the number of deployed sensors increases, total network energy consumption increases correspondingly, a critical factor to consider for long-term, sustainable forest fire monitoring.

These results validate that the proposed method manages network load and energy consumption while ensuring low-latency emergency fire notifications, maintaining operational efficiency in diverse forest conditions.

In sparse forests with clear line-of-sight, sensors consume less energy compared to those deployed in dense forest environments. This is due to the damping and attenuation effects caused by dense foliage, moisture, and terrain obstacles, which increase the transmission power required for maintaining connectivity and data delivery during forest fire monitoring.

Leveraging IEEE 802.11af technology within the CRSN framework enables opportunistic access to licensed channels without spectrum fees, using cognitive radio capabilities for dynamic spectrum sensing and allocation. This approach significantly enhances forest fire monitoring by ensuring real-time, cost-effective, and energy-efficient emergency alerting using white space channels under diverse forest conditions.

Wireless sensor networks, widely utilized globally for environmental monitoring, find an effective application in forest fire detection using the proposed cognitive radio FDMA structure, ensuring sustainability and scalability for real-world deployment.

In the sphere of forest fire monitoring systems, IEEE 802.11af technology plays a crucial role in enabling reliable, real-time environmental data tracking. Within cognitive radio sensor networks (CRSNs), setting data transmission priorities based on urgency is essential to ensure that critical fire detection alerts are delivered with minimal delay during emergencies.

The validity of the proposed forest fire monitoring network has been demonstrated graphically using results from both MATLAB-based simulations and analytical models. These results confirm the system’s ability to maintain low-latency, energy-efficient, and reliable communication during forest fire detection and alerting operations. It has been determined that a cognitive radio sensor network based on IEEE 802.11af technology can effectively transmit urgent fire detection data by dynamically prioritizing packets using priority-based monitoring. This system operates without incurring any spectrum costs by leveraging television white space channels opportunistically, making it a cost-effective, scalable, and sustainable solution for forest fire monitoring and emergency notification.

*Impact of Complex Terrains*: The variations in terrain, such as valleys, hills, and uneven ground, can significantly affect signal propagation in forest environments. While the current simulation incorporates path loss models that approximate dense forest conditions, it does not explicitly model terrain features like elevation changes or obstacles.

*Dynamic Environmental Factors*: The work currently includes parameters such as path loss coefficient and coverage distance, which can be tuned to reflect varying environmental conditions, including changes in foliage density. However, a more comprehensive long-term evaluation is necessary to account for seasonal variations in vegetation, humidity, and other factors that influence propagation. These can be incorporated in future work using sensor data such as humidity, temperature, and foliage density to adaptively adjust spectrum access strategies and transmission parameters.

**Fig. 12:**
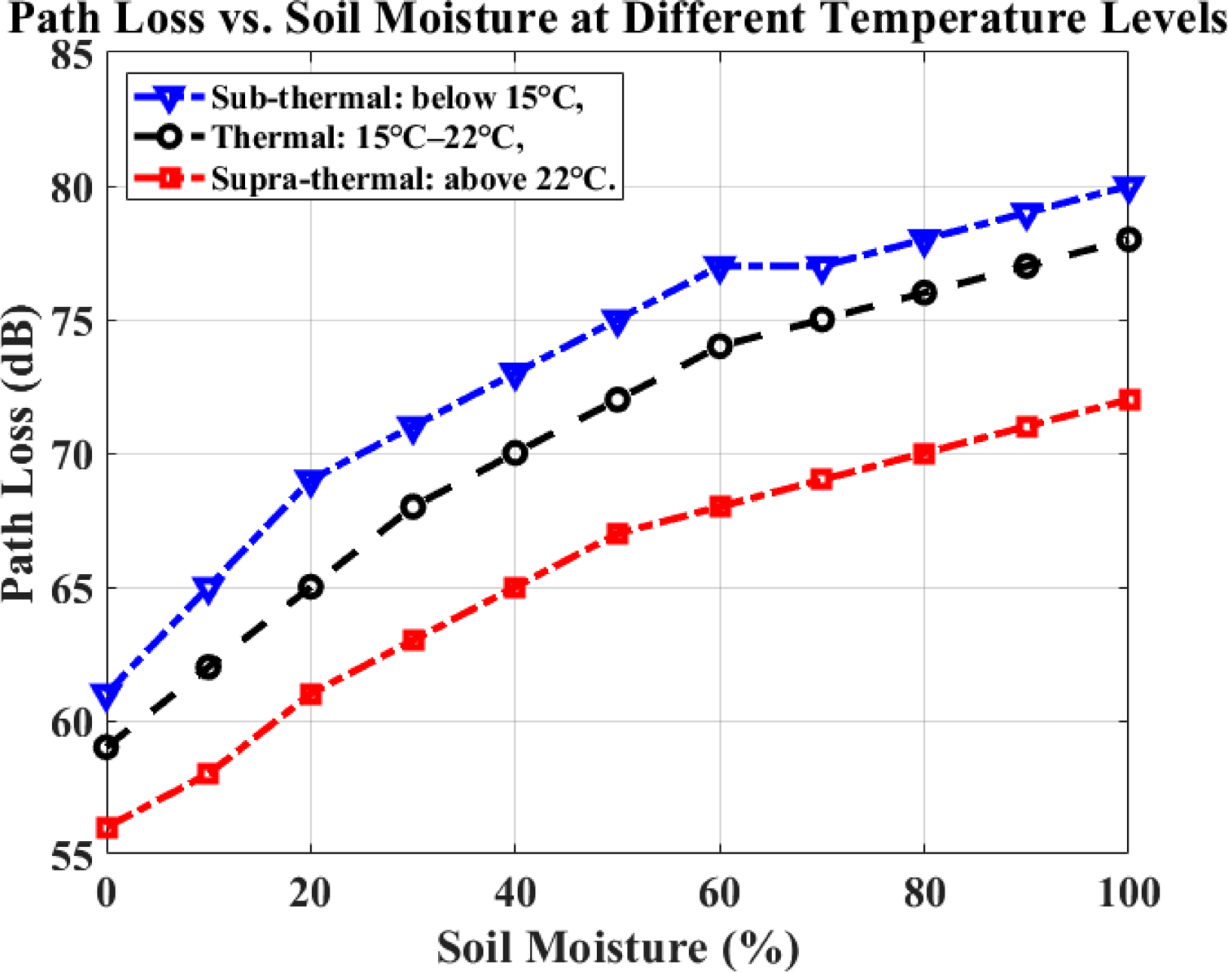
Soil moisture versus path loss.

Figure 12 shows the path loss results under varying temperature conditions based on soil moisture in a forest environment. This analysis enhances the system’s generalization capabilities across diverse forest terrains and weather conditions critical for fire monitoring. Soil moisture varies from 0 to 100%, while path loss ranges from 56 to 80 dB.

Temperature conditions are categorized as:

- Sub-thermal: below 15 °C,
- Thermal: 15 °C–22 °C,
- Supra-thermal: above 22 °C.
  - For example, in a forest area with 50% soil moisture under temperature conditions, the path loss is approximately 75 dB. These results confirm the proposed system’s resilience and environmental adaptability under different forest conditions, ensuring reliable communication during forest fire monitoring. Table 3 shows the performance of the proposed method under different temperatures.

**Table 3:** shows the performance of the proposed methodology.

Soil moisture (%)	Sub-thermal: below 15 °C	Thermal: 15 °C–22 °C	Supra-thermal: above 22 °C	Remarks
0	Lowest baseline path loss among all moisture levels, but higher than hot conditions	Slightly lower than cold	Lowest	Dry soil, minimal attenuation; temperature effect is small
25	Increases path loss due to moisture absorption	Increases but less than cold	Increases, but still the lowest	Early moisture accumulation affects cold more
50	~ 75 dB	~ 72 dB	~ 67 dB	Clear temperature separation in attenuation
75	Higher than 50%	Moderate increase	Smaller increase	High attenuation risk in cold
100	Maximum path loss (cold > moderate > hot)	–	–	Fully saturated soil strongly attenuates RF signals

**Fig. 13:**
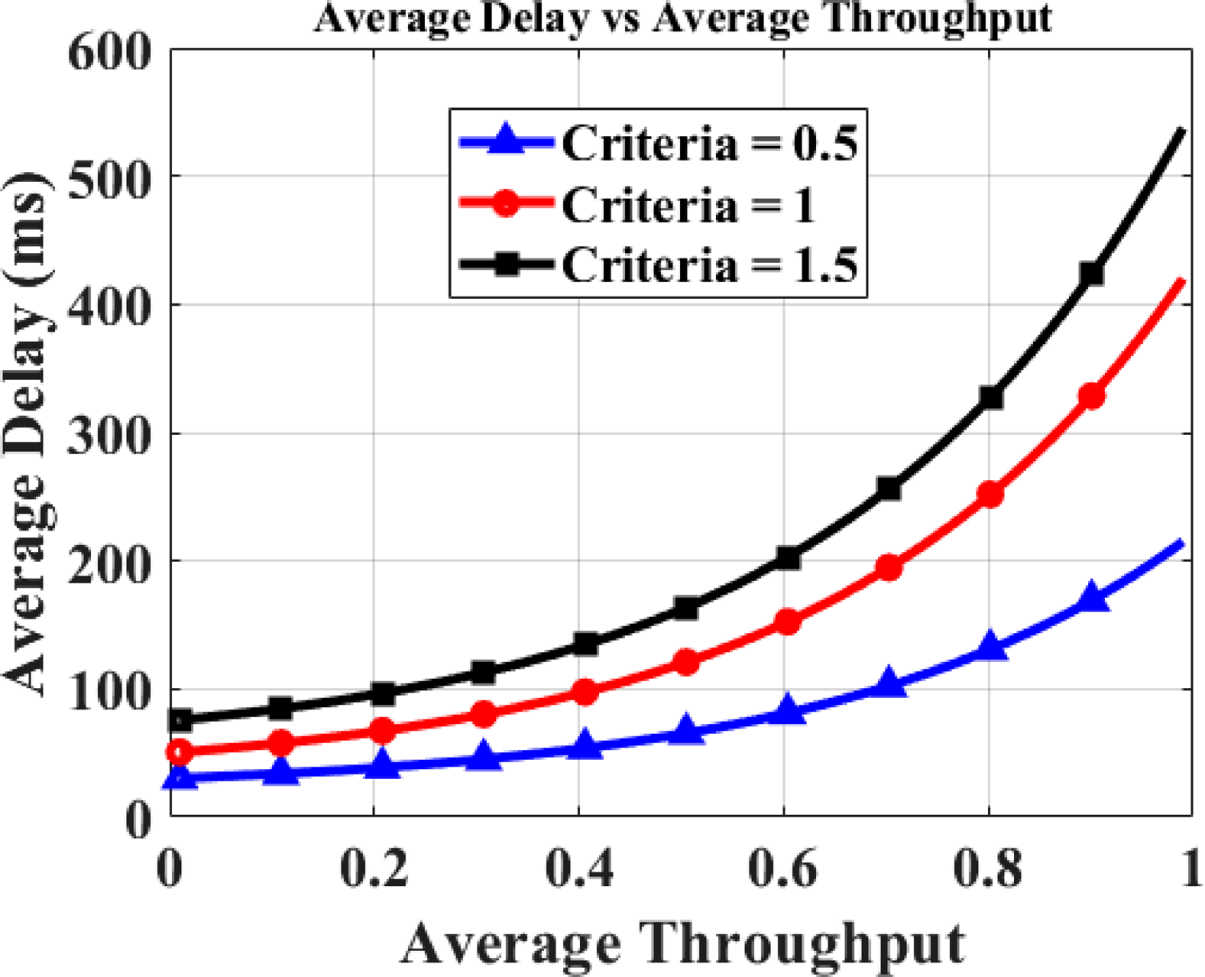
Average throughput versus delay.

Figure 13 shows the impact of duration criteria on average throughput and delay within the forest fire CRSN, and the data is tabulated in Table 4. Different criteria are evaluated to enhance the flexibility of system design for priority-based emergency notification.

- Average throughput varies from 0 to 1, while average delay ranges from 0 to 600 ms.
- Three criteria are considered: 0.5, 1, and 1.5.

**Table 4:** Average throughput and delay analysis.

Duration criteria	Average throughput	Average delay (ms)	Observation
0.5	0.8	130	Prioritizes low latency for rapid fire alerts, sacrificing some throughput
1.0	0.85	275	Balanced trade-off between throughput and latency
1.5	0.9	423	Achieves the highest throughput, but increases delay due to queuing of lower-priority packets

For example:

For 0.5, the system achieves an average throughput of 0.8 with an average delay of 130 ms, prioritizing low-latency fire alerts.

For 1.5, the system achieves an average throughput of 0.9 with an average delay of 423 ms, reflecting higher throughput with increased queuing for lower priority packets.

These results validate that adjusting the criteria enables a tunable trade-off between throughput and latency in the proposed forest fire CRSN, providing system designers the flexibility to optimize for rapid fire alerts or high-throughput monitoring based on deployment requirements.

## Conclusion

In this work, we proposed a cognitive radio sensor network (CRSN) based on IEEE 802.11af technology for forest fire monitoring with adaptive priority-based data transmission. Within the network, CRSNs, Cognitive Nodes (CNs), and a Cognitive Base Station (CBS) communicate opportunistically over television white spaces using FDMA, ensuring no harmful interference with licensed services. Sensor nodes deployed in forest environments collect data, which is combined with priority classes (level 1, level 2, level 3) according to the urgency of the information.

To prevent queue starvation during emergencies, an adaptive priority adjustment mechanism was incorporated, dynamically elevating the priority of critical data, such as sudden spikes in temperature that may indicate forest fires. By integrating adaptive priority management with FDMA for spectrum allocation, our work addresses the critical challenges of timely data delivery and energy-efficient operation in forest monitoring networks. The network was modeled analytically and simulated in MATLAB, with results demonstrating the feasibility, effectiveness, and energy efficiency of the system, all achieved without incurring spectrum costs. Path loss and energy consumption analysis under different moisture and temperature conditions will further support the system’s generalizability across diverse forest environments, ensuring scalable, resilient, and cost-effective forest fire monitoring.

The proposed framework provides a cost-effective solution for early fire detection, potentially mitigating environmental and economic losses. While the current evaluations are simulation-based and rely on spectrum database guidance (LAS and CLB), the system design is flexible and can accommodate environmental variability in future deployments.

As future work, we plan to implement the network in real forest environments to validate its performance under diverse conditions, such as valleys, hills, and variations in soil moisture, temperature, and vegetation density. Additionally, energy consumption analysis across different sensor deployments and integration with 5G-based low-latency frameworks will be explored. The simulation models of the integrating adaptive priority management developed in this work will serve as a foundation for these real-world experiments, ensuring scalable, resilient, and effective forest fire monitoring.

## Data Availability

The datasets used and/or analysed during the current study available from the corresponding author on reasonable request.
